# The correlation between LAG-3 expression and the efficacy of chemoimmunotherapy in advanced biliary tract cancer

**DOI:** 10.1007/s00262-024-03878-0

**Published:** 2025-01-03

**Authors:** Cheng-Yu Tang, Yi-Ting Lin, Yi-Chen Yeh, Shin-Yi Chung, Yu-Chan Chang, Yi-Ping Hung, San-Chi Chen, Ming-Huang Chen, Nai-Jung Chiang

**Affiliations:** 1 Division of Medical Oncology, Department of Oncology, Taipei Veterans General Hospital, No. 201, Sec. 2, Shipai Road, Beitou District, Taipei, 112201 Taiwan; 2 School of Medicine, National Yang Ming Chiao Tung University, Taipei, Taiwan; 3 Department of Pathology and Laboratory Medicine, Taipei Veterans General Hospital, Taipei, Taiwan; 4 Institute of Biomedical Informatics, National Yang Ming Chiao Tung University, Taipei, Taiwan; 5 Genomics Research Center, Academia Sinica, Taipei, Taiwan; 6 Department of Biomedical Imaging and Radiological Sciences, National Yang Ming Chiao Tung University, Taipei, Taiwan; 7 Institute of Clinical Medicine, National Yang Ming Chiao Tung University, Taipei, Taiwan; 8 National Institute of Cancer Research, National Health Research Institutes, Tainan, Taiwan

**Keywords:** Cholangiocarcinoma, Immune checkpoint inhibitors, LAG-3 protein, PDCD1 protein, CD274 protein

## Abstract

In our previous phase II T1219 trial for advanced biliary tract cancer (ABTC), the combination of nivolumab with modified gemcitabine and S-1 exhibited promising efficacy, while the programmed-death-ligand-1 (PD-L1) expression did not predict chemoimmunotherapy efficacy. Lymphocyte-activation-gene-3 (LAG-3), a negative immune checkpoint, is frequently co-expressed with PD-L1. This study assessed the predictive value of LAG-3 expression in ABTC patients who received chemoimmunotherapy. We analyzed 44 formalin-fixed ABTC samples using immunohistochemical staining for PD-L1 and LAG-3 and correlated them with the clinical efficacy of chemoimmunotherapy. Digital spatial profiling was conducted in selected regions of interest to examine immune cell infiltration and checkpoint expression in six cases. Three public BTC datasets were used for analysis: TCGA-CHOL, GSE32225, and GSE132305. LAG-3 positivity was observed in 38.6% of the ABTC samples and was significantly correlated with PD-L1 positivity (*P* < 0.001). The objective response rate (ORR) was significantly higher in LAG-3-positive tumors than in LAG-3-negative tumors (70.6% vs. 33.3%, *P* = 0.029). The LAG-3 expression level was associated with an increased ORR (33%, 58%, and 100% for LAG-3 < 1%, 1–9%, and ≥ 10%, respectively; *P* = 0.018) and a deeper therapeutic response (20.1%, 38.6%, and 57.6% for the same respective groups; *P* = 0.04). LAG-3 expression is positively correlated with the expression of numerous immune checkpoints. Enrichment of CD8^+^ T cells was observed in LAG-3-positive BTC, indicating that LAG-3 expression may serve as a biomarker for identifying immune-inflamed tumors and predicting the therapeutic response to chemoimmunotherapy in ABTC.

## Introduction

Biliary tract cancer (BTC) comprises intrahepatic and extrahepatic cholangiocarcinomas (IHCC and EHCC), gallbladder cancer (GBC), and ampulla of Vater cancer (AVC). Approximately 60–70% of BTC cases are unresectable and advanced at diagnosis; gemcitabine plus cisplatin (GC) is the standard first-line treatment, offering a median overall survival (OS) of 11.7 months [1, 2]. In Japan, Korea, and Taiwan, the use of gemcitabine plus S-1 (GS) as an alternative first-line option was demonstrated to be non-inferior to GC in a phase 3 JCOG 1113 study [3]. Despite these advances, the prognosis for patients with advanced BTC (ABTC) remains unsatisfactory; moreover, treatment strategies continue to evolve 10 years after the pivotal ABC-02 trial, emphasizing the ongoing need for novel therapeutic approaches [4]. Advances in next-generation sequencing have revealed the genomic landscape of BTC, enabling the development of targeted therapies that can significantly alter the treatment and prognosis of patients with advanced BTC (ABTC). Isocitrate dehydrogenase 1 (IDH1) and fibroblast growth factor receptor (FGFR) inhibitors have been approved by the U.S. Food and Drug Administration (U.S. FDA) to treat refractory ABTC harboring *IDH1* mutation or *FGFR2* fusion [5]. However, the prognosis of patients with ABTC without actionable mutations has not improved in the past decade.

Immune evasion is a hallmark of cancer development. IHCC is characterized by desmoplastic stroma with abundant cancer-associated fibroblasts and tumor-associated macrophages that contribute to the immunosuppressive tumor microenvironment by promoting immune tolerance, tumor growth, and metastasis [6]. Job et al. classified IHCC into four immune subtypes based on gene expression signatures and immunohistochemistry (IHC), with the immune-inflamed subtype showing significant T-cell infiltration, activated immune checkpoint pathways, and a better prognosis than the immune-desert, myeloid, and mesenchymal subtypes [7]. Immune checkpoint inhibitors (ICIs), including anti-programmed-death-1 (PD-1), anti-programmed-death-ligand-1 (PD-L1, also called CD274), and anti-cytotoxic-T-lymphocyte-associated-protein-4 (CTLA-4) monoclonal antibodies, have shown durable antitumor activity in various cancer types by reactivating the ability of immune cells to recognize and eliminate tumor cells [8]. Emerging evidence supports the clinical benefit of ICIs in ABTC, leading to their approval by the U.S. FDA [9, 10]. Combining GC with pembrolizumab or durvalumab has emerged as a new standard first-line treatment for ABTC, achieving 26–29% response rates. However, PD-L1 expression lacks predictive value for identifying ICI-sensitive subgroups of ABTC, underscoring the need for further research to elucidate the tumor immune microenvironment and identify predictors of ICI response [9, 10]. Studies have investigated DNA damage repair pathways in BTC, particularly BRCA1/2 mutations, which may be linked to higher tumor mutation burden, microsatellite instability, and mismatch repair deficiency, suggesting a greater likelihood of response to immunotherapy [11]. Additionally, studies have emphasized the importance and variety of immune-related adverse events in patients treated with ICIs, which can impact treatment outcomes and quality of life [12–14].

Lymphocyte activation gene 3 (LAG-3) is a transmembrane immune checkpoint protein with a 20% structural homology to CD4 and is expressed on T cells, natural killer (NK) cells, and dendritic cells. LAG-3 inhibits effector T cell activation by interacting with major histocompatibility complex (MHC) class II molecules and downregulating T cell receptor signaling [15]. LAG-3 is highly co-expressed with PD-1 in tumor-infiltrating CD4^+^ and CD8^+^ T lymphocytes, and dual inhibition of LAG-3 and PD-1 has demonstrated synergistic antitumor activity in preclinical studies [16]. Opdualag, the combination of anti-PD-1 (nivolumab) and anti-LAG-3 (relatlimab) antibodies has demonstrated clinical efficacy in melanoma. It shows a higher pathological complete response rate in early-stage melanoma and improved progression-free survival (PFS) in advanced cases, leading to its U.S. FDA approval [17, 18].

The role of LAG-3 in ABTC remains unclear. Therefore, our study aimed to investigate the clinicopathological correlation between LAG-3 and PD-L1 expression, the tumor immune microenvironment, and its predictive value in chemoimmunotherapy-treated ABTC, primarily using tissue samples from the T1219 phase 2 trial, in which nivolumab combined with modified gemcitabine and S-1 was evaluated as a frontline treatment for ABTC [19].

## Materials and methods

### Study design and participants

The trial design and eligibility criteria for T1219 have been previously described [19]. The T1219 trial was a single-arm, multicenter, phase II trial in Taiwan that recruited patients with previously untreated, histologically confirmed, locally advanced, or metastatic BTC. The study regimen consisted of intravenous infusion of fixed-dose 240 mg nivolumab and 800 mg/m^2^ gemcitabine on day 1, plus oral administration of S-1 on days 1 to 10 in a 2-week cycle. Treatment was administered until disease progression, intolerable toxicity, withdrawal of consent, or any other reason. The study's primary endpoint was the objective response rate (ORR), assessed using radiographic imaging according to the Response Evaluation Criteria in Solid Tumors (RECIST) version 1.1. The objective response was confirmed using two successive imaging studies. The secondary endpoints included long-term disease control rate (DCR), PFS, and OS. Long-term DCR was defined as a complete, partial, or stable disease for at least 12 weeks. PFS was calculated as the duration from the first dose to either the first documented disease progression, death, or were censored. Overall survival (OS) was defined as the time from study entry to either death or censoring. The cutoff for continuous survival follow-up was set for August 2024.

The trial protocol and post hoc analyses were approved by the respective institutional review boards of all participating institutes and registered with ClinicalTrials.gov (NCT04172402). Written informed consent was obtained from all patients prior to their participation in the study. This study was conducted in accordance with the Declaration of Helsinki and the International Conference on Harmonization Good Clinical Practice guidelines.

### IHC staining of PD-L1 and LAG-3

**Fig. 1:**
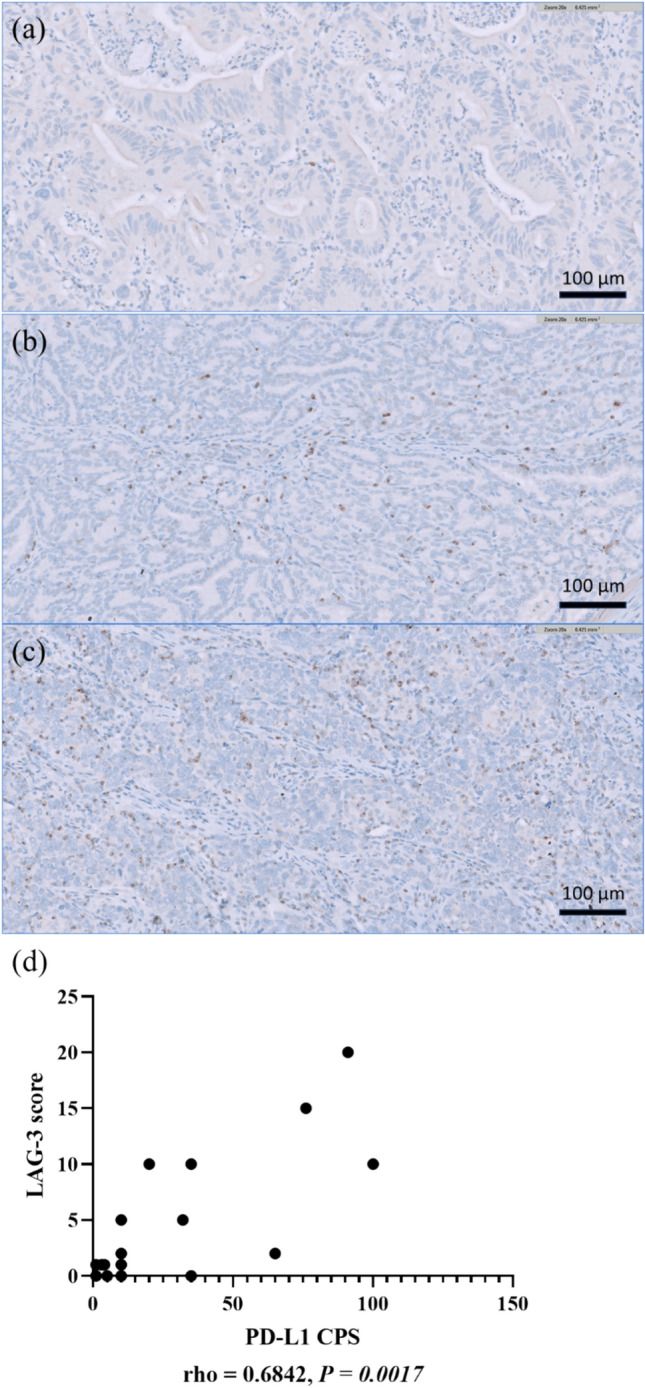
LAG-3 expression on immune cells evaluated by immunohistochemistry. Note. **a** LAG-3 positive at 1% (high-power view at magnifications of ×20), **b** LAG-3 positive at 5% (high-power view at magnifications of ×20), **c** LAG-3 positive at 10% (high-power view at magnifications of ×20), **d** Correlation analyses between the percentage of LAG-3 and PD-L1 CPS score by Spearman’s test

Archival paraffin-embedded tumor samples were obtained prior to treatment and were used for PD-L1 IHC staining by Dako 22C3 pharmDx assay (Dako North America) and LAG-3 IHC staining by antibody (clone 17B4, Enzo Life Sciences). An independent pathologist at the central laboratory evaluated the results. The PD-L1 combined positive score (CPS) was defined as the percentage of PD-L1-stained cells, including tumor cells, lymphocytes, and macrophages, divided by the total number of viable tumor cells and multiplied by 100. PD-L1 positivity was defined as PD-L1 CPS ≥ 1%. LAG-3 expression was defined as the percentage of immune cells with a positive punctate, membrane, and/or cytoplasmic staining relative to all nucleated cells in the tumor region containing at least 100 viable tumor cells (Fig. 1). Immune cells with LAG-3 ≥ 1% were considered LAG-3-positive and further stratified into subgroups based on the percentage of LAG-3 expression.

### GeoMx digital spatial profiler

The GeoMx Digital Spatial Profiler (DSP) from NanoString Technologies is used in immuno-oncological research to profile RNA and protein expression in spatial contexts. Our workflow involved comprehensive whole-slide staining, employing assay probes along with four fluorescent markers: CD20 (yellow) for B cells, CD3E (red) for T cells, KRT18 (green) for tumor cells, and DNA (blue) for nuclei, followed by precise imaging for targeted selection of tissue regions for profiling. Masking methodologies facilitated the optional profiling of multiple tissue compartments or distinct cell types within each region. We performed a comprehensive spatial analysis of six cholangiocarcinoma samples. We defined 30 regions of interest (ROIs) as lymphocyte-infiltrated regions based on the expression levels of the fluorescent markers, particularly higher T-cell expression, and conducted further analyses.

### Public dataset exploration

Expression of CD274, LAG-3 and the genes for programmed cell death 1 (PDCD1), programmed cell death 1 ligand 2 (PDCD1LG2), CTLA4, hepatitis A virus cellular receptor 2 (HAVCR2, also known as TIM-3), T cell immunoreceptor with immunoglobulin and immunoreceptor tyrosine-based inhibitory motif domains (TIGIT), CD27, CD28, CD48, CD70, CD80, CD86 and inducible T cell co-stimulator (ICOS) in individual patients from the profiles TCGA-CHOL, GSE32225 and GSE132305 and the distribution in different pathologic stage-related information were downloaded from the Xena Functional Genomics Explorer (http://xenabrowser.net).

### Correlation and prediction between the target gene and immune populations

We used the Tumor and Immune System Interaction Database (TISIDB), an online tool integrating genomic, transcriptomic, and clinical data from TCGA dataset, to explore the correlation between LAG-3 expression and other immune checkpoints and the abundance of tumor-infiltrating immune cells in the TCGA-CHOL cohort, including CD8^+^ T cells, CD4^+^ T cells, regulatory T cells, macrophages, myeloid-derived suppressor cells, and NK cells [20].

### Statistical and data analysis

The Kaplan–Meier method was used to determine the median PFS and OS with 95% confidence intervals (CI), and the log-rank test was used to compare the PFS and OS differences between subgroups. ORR was compared using Fisher’s exact test. The correlation between PD-L1 and LAG-3 expression was calculated using Spearman’s and Fisher’s exact tests. The concordance between LAG-3 and PD-L1 positivity was calculated using Cohen’s kappa test. The correlation between LAG-3 and other immune checkpoints and immune cells was analyzed using the Spearman’s test. All analyses were performed using GraphPad Prism (version 9.0; GraphPad Inc.) and SPSS software version 22 (IBM Corp., Armonk, NY, USA). A two-sided *P* < 0.05 was considered statistically significant.

## Results

### Patient characteristics

A total of 44 pretreated samples were analyzed for LAG-3 using IHC staining. Baseline demographics are presented in Table 1. The median age of the participants was 67 years (range 30–80 years), and 54% of the study population were women. Metastatic disease was observed in 84% of the participants. The distribution of IHCC, EHCC, GBC, and AVC within the study group was 59%, 25%, 11.4%, and 4.5%, respectively. The ORR and long-term DCR rates were 47.7% and 77%, respectively (Table 2). The median OS and PFS were 15.4 and 7.9 months, respectively.

**Table 1:** Demographic difference between LAG-3-positive and LAG-3-negative subgroup

Characteristic	All (N = 44)	LAG-3-positive (N = 17)	LAG-3-negative (N = 27)	*P* value
Age				
Median (range), year	67 (30–80)	67 (30–80)	68(35–80)	0.407
Sex (%)				
Male	20 (45)	8 (47)	12 (44)	> 0.99
Female	24 (55)	9 (53)	15 (56)	
Stage (%)				
II	1 (2)	0 (0)	1 (4)	NA
III	2 (5)	0 (0)	2 (7)	
IIIB	4 (9)	2 (12)	2 (7)	
IVA	7 (16)	3 (18)	4 (15)	
IVB	30 (68)	12 (70)	18 (67)	
Primary tumor site (%)				
Intrahepatic bile duct	26 (59)	9 (53)	17 (63)	NA
Extrahepatic bile duct	11 (25)	5 (29)	6 (22)	
Gallbladder	5 (11)	1 (6)	4 (15)	
Ampulla of vater	2 (5)	2 (12)	0 (0)	
PD-L1 expression (%)				
CPS ≥ 1	18 (41)	14 (82)	4 (15)	< 0.001
CPS < 1	26 (59)	3 (18)	23 (85)	

The tumor stage was based on the eighth edition of the American Joint Committee on Cancer

Abbreviations: LAG-3, lymphocyte activation gene 3; PD-L1, programmed-death-ligand-1; CPS, combined positive score; NA, not applicable

**Table 2:** Response rate in this study

Best overall response	N = 44 (%)
Complete response	0 (0)
Partial response	21 (47.7)
Stable disease	4 (9.1)
Stable disease ≥ 12 weeks	13 (29.5)
Progressive disease	5 (11.4)
Not evaluated	1 (2.3)
Objective response	21 (47.4)
Long-term disease control	34 (77.3)

Objective response includes complete response and partial response. Long-term disease control includes complete response, partial response and stable disease ≥ 12 weeks

### Correlation between LAG-3 and PD-L1 expression

The LAG-3 expression was observed in 38% of the tumors, with positivity rates of 34.6, 45.5, 20, and 100% in IHCC, EHCC, GBC, and AVC, respectively. No significant differences were observed in age, sex, or stage between the LAG-3-positive and LAG-3-negative groups. The proportion of PD-L1 CPS ≥ 1 tumors were 41% across the entire cohort; 82% of LAG-3-positive tumors were PD-L1 CPS ≥ 1, whereas 15% of LAG-3-negative tumors presented PD-L1 CPS ≥ 1. LAG-3 expression was significantly correlated with PD-L1 expression (*P* < 0.001), demonstrating substantial concordance (kappa = 0.668, 95% confidence interval: 0.443–0.893). Spearman’s correlation analysis indicated a moderately positive correlation (r = 0.684, *P* = 0.0017) between the percentage of LAG-3 expression and the PD-L1 CPS score (Fig. 1d).

**Fig. 2:**
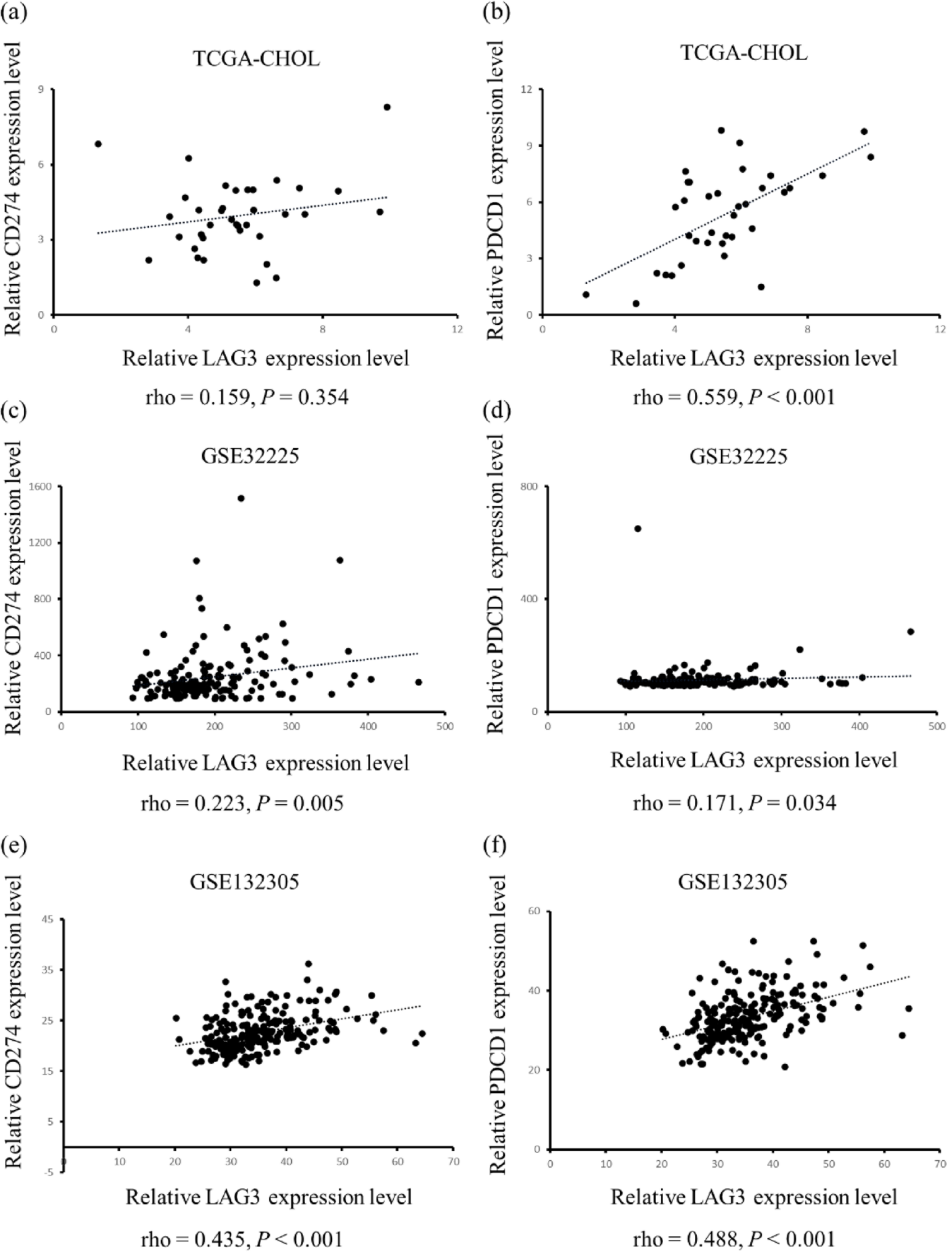
Correlation analyses between LAG-3 and PD-L1, PD-1 expression in public BTC datasets. Note. Correlation analyses were conducted by RNA sequencing data extracted from three public BTC datasets: TCGA-CHOL, GSE32225, and GSE132305. The correlations between LAG-3 and PD-L1 are presented in panels **a**, **c**, and **e** corresponding to TCGA-CHOL, GSE32225, and GSE132305 datasets, respectively. Similarly, the associations between LAG-3 and PD-1 are depicted in panels **b**, **d**, and **f** and aligned with the same datasets

We further confirmed the relationship among LAG-3, PDCD1, and CD274 in three independent cohorts. The expression of LAG-3 positively correlated with that of CD274 in GSE32225 (r = 0.223, *P* = 0.005, Fig. 2c) and GSE132305 cohorts (r = 0.435, *P* < 0.001, Fig. 2e); however, there was no correlation in the TCGA-CHOL cohort (r = 0.159, *P* = 0.354, Fig. 2a). The correlation between LAG-3 and PDCD1 was significant in all three cohorts (TCGA-CHOL: r = 0.559, *P* < 0.001; Figure 2b; GSE32225: r = 0.171, *P* = 0.034; Fig. 2d; GSE132305: r = 0.488, *P* < 0.001; Fig. 2f) [21–23].

### Association between LAG-3 and PD-L1 expression in predicting ICI treatment outcomes

**Fig. 3:**
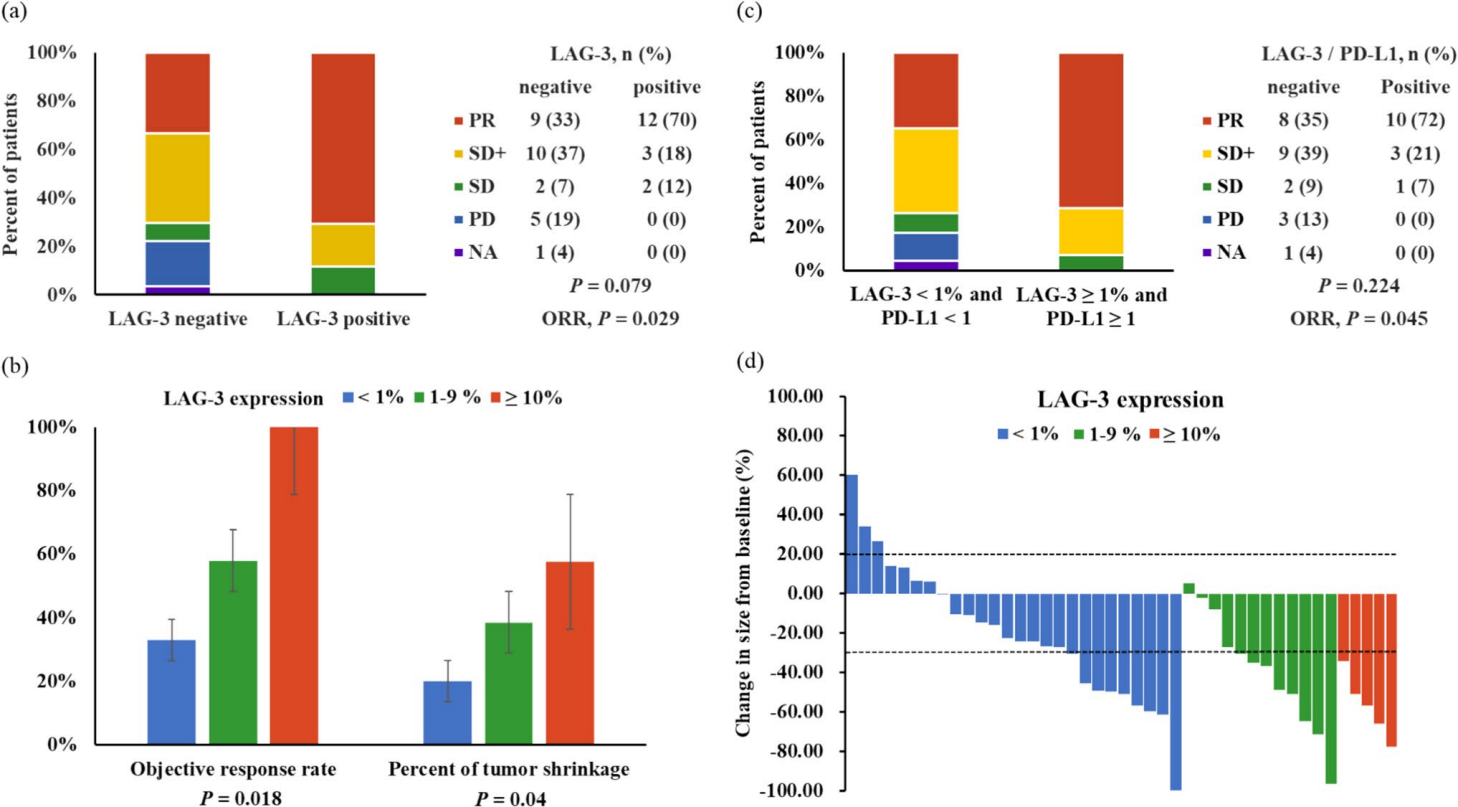
Analyses of response rates and tumor shrinkage across subgroups defined by LAG-3 and PD-L1 CPS. Note. **a** ORR between LAG-3-positive and LAG-3-negative subgroups, **b** ORR and percentage of tumor shrinkage across three subgroups categorized by LAG-3 expression levels: less than 1%, between 1-9%, and 10% or greater, **c** ORR comparison in subgroups stratified by concurrent positivity or negativity for LAG-3 and PD-L1, **d** Waterfall plot illustrating the percentage change in tumor size within subgroups defined by LAG-3 expression levels: less than 1%, between 1–9%, and 10% or greater. Abbreviations: PR, partial response; SD^+^, stable disease ≥ 12 weeks; SD, stable disease; PD, progressive disease; NA, not evaluated

**Fig. 4:**
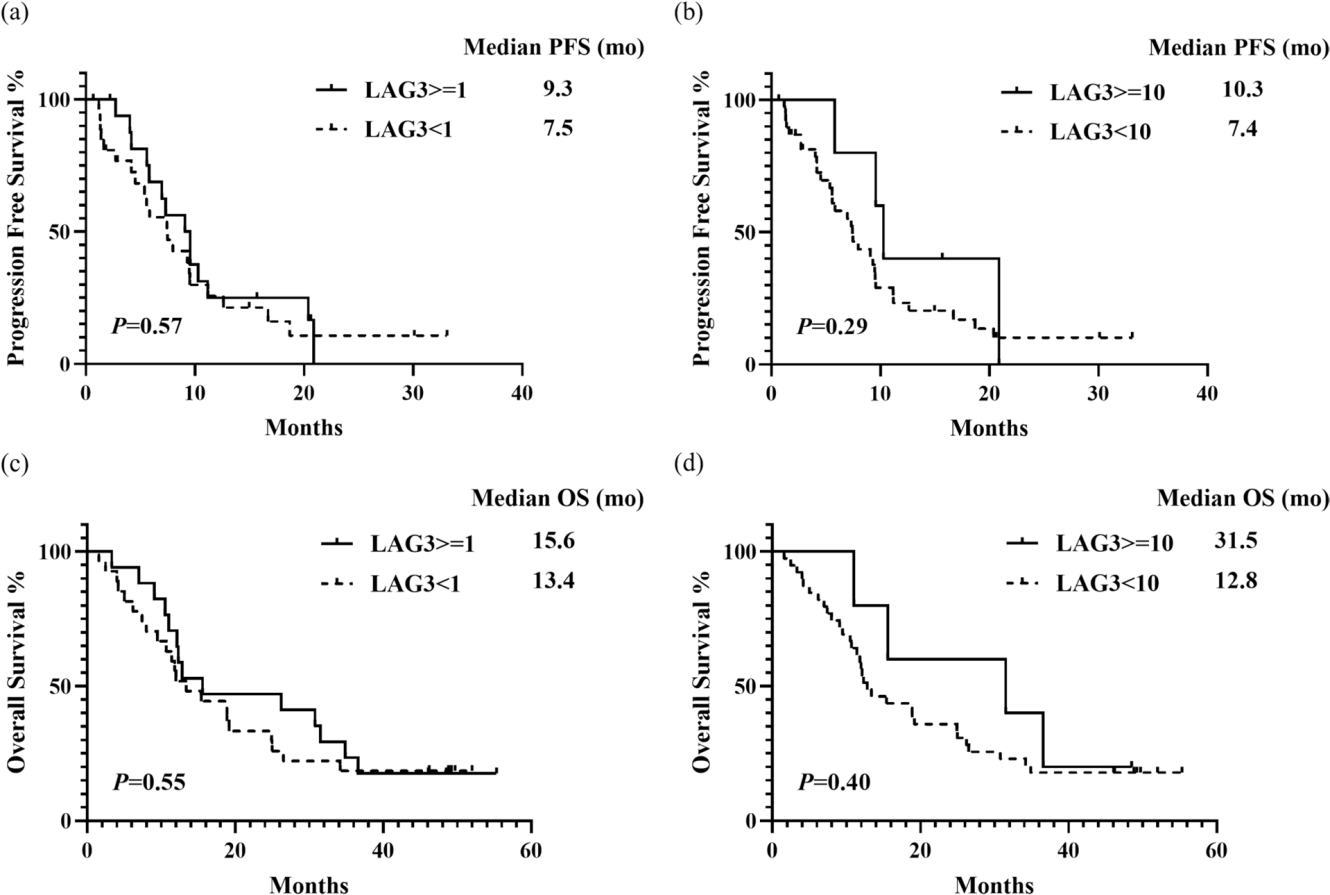
Median PFS and OS across subgroups defined by LAG-3 expression levels. Note. **a** PFS of subgroups with LAG-3 expression levels of less than 1% and 1% or greater, **b** PFS of subgroups with LAG-3 expression levels of less than 10% and 10% or greater, **c** OS of subgroups with LAG-3 expression levels of less than 1% and 1% or greater, **d** OS of subgroups with LAG-3 expression levels of less than 10% and 10% or greater

LAG-3 positivity was significantly correlated with a higher ORR of 70.6% compared to 33.3% in the LAG-3-negative group (*P* = 0.029, Fig. 3A), a numerically higher long-term DCR of 88.2% vs. 70.3% (*P* = 0.271), and a greater mean percentage of tumor shrinkage (−48.7% vs. −23.6%, *P* = 0.014). Patients were categorized into subgroups based on LAG-3 expression levels: < 1%, 1–9%, and ≥ 10%. Increased ORR and tumor shrinkage were observed at higher LAG-3 expression levels (Fig. 3b and d). A trend was noted toward longer PFS and OS in subgroups with LAG-3 expression ≥ 1% and ≥ 10% compared to those with expression < 1% and < 10%, although these differences were not statistically significant owing to the small sample size (Fig. 4).

In our previous study, PD-L1 expression alone did not demonstrate a predictive value for ORR, long-term DCR, PFS, or OS [19]. By incorporating LAG-3 and PD-L1 expressions into the analysis, we observed that ORR was higher in tumors with both LAG-3 expression ≥ 1% and PD-L1 CPS ≥ 1 compared to those with negative expression for both markers (72% vs. 35%, *P* = 0.045, Fig. 3c). Additionally, this group had a greater percentage of tumor shrinkage (−42.5% vs. −23.6%, *P* = 0.083). However, no significant differences were observed in the long-term DCR, median PFS, or median OS between the groups.

### Correlation between LAG-3, the other immune checkpoint molecules and tumor-infiltrated immune cells

**Fig. 5:**
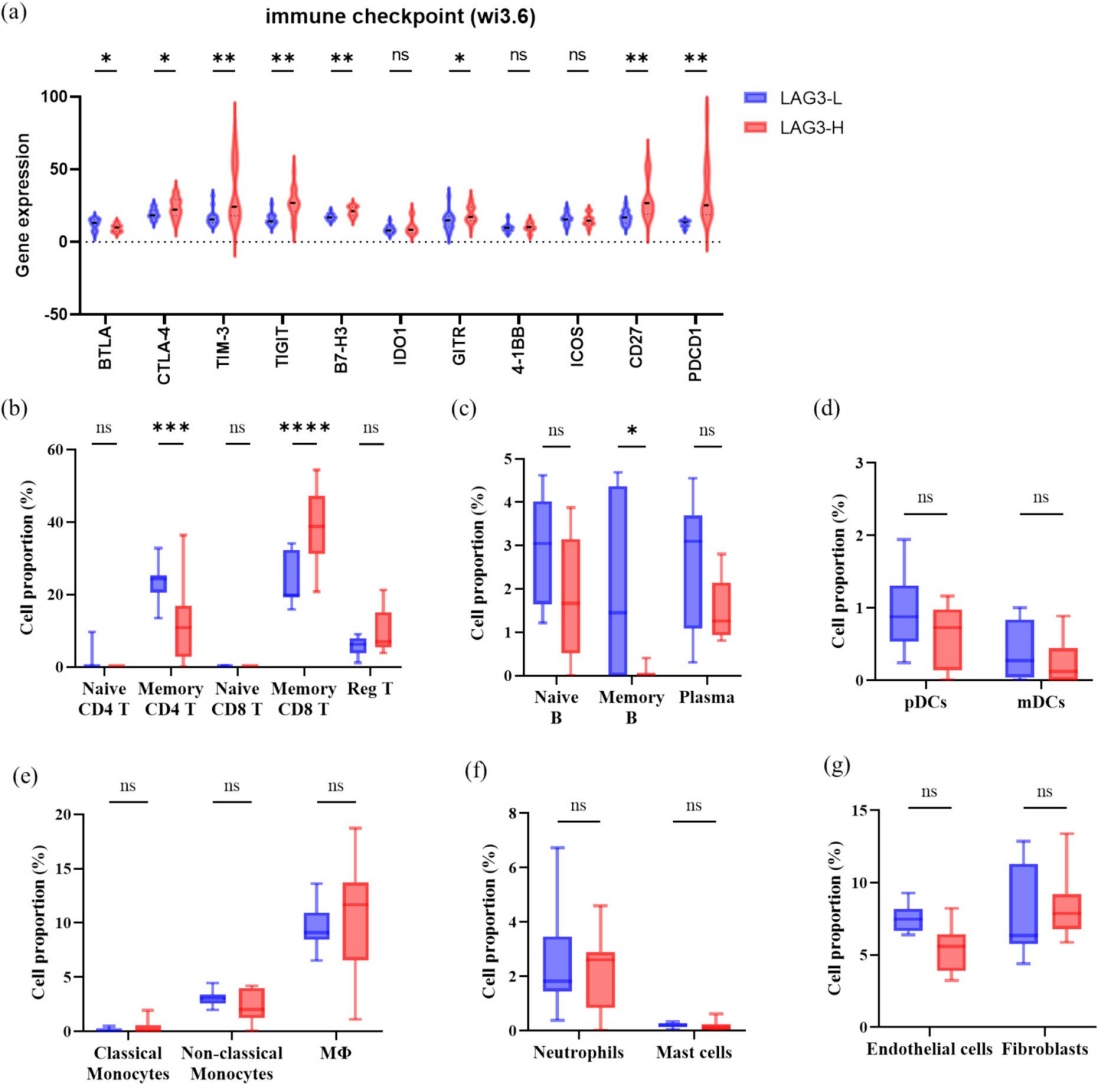
Comparison analyses of expression of immune checkpoints and immune cells between subgroups with high and low LAG-3 expression (RNA sequencing) in our BTC cohort. Note. Analysis of **a** immune checkpoints, **b** CD4^+^ and CD8^+^ T cells, **c** B cells, **d** dendritic cells, **e** monocytes, **f** neutrophils and macrophages, and **g** endothelial cells and fibroblasts using the GeoMx Digital Spatial Profiler. Abbreviations: Reg T, regulatory T cell; Plasma, plasma cell; pDC, plasmacytoid dendritic cell; mDC, myeloid dendritic cell; MΦ, macrophage

We conducted RNA sequencing of 30 ROIs, which were defined as lymphocyte-infiltrated areas, and categorized them into groups with high and low LAG-3 expression based on the median level of LAG-3 (Fig. 5). High LAG-3 expression is associated with elevated levels of several immune checkpoints, including PDCD1, CTLA-4, TIM3, TIGIT, B7 homolog 3 protein (B7-H3), glucocorticoid-induced TNFR-related protein (GITR), and CD27, along with reduced expression of B- and T-lymphocyte attenuators (BTLA), indicating that these immune checkpoints are highly relevant.

Furthermore, of the 30 ROIs from 6 patients, 18 ROIs from four patients received chemoimmunotherapy and were clustered into high and low LAG-3 expression groups. High LAG-3 expression was associated with a high proportion of memory CD8^+^ T cells (Fig. 5). Conversely, memory CD4^+^ and B cells were more abundant in tumors with low LAG-3 expression levels. No significant differences were observed in the proportions of regulatory T cells, dendritic cells, neutrophils, mast cells, monocytes, or fibroblasts between tumors with high and low LAG-3 expression levels.

**Fig. 6:**
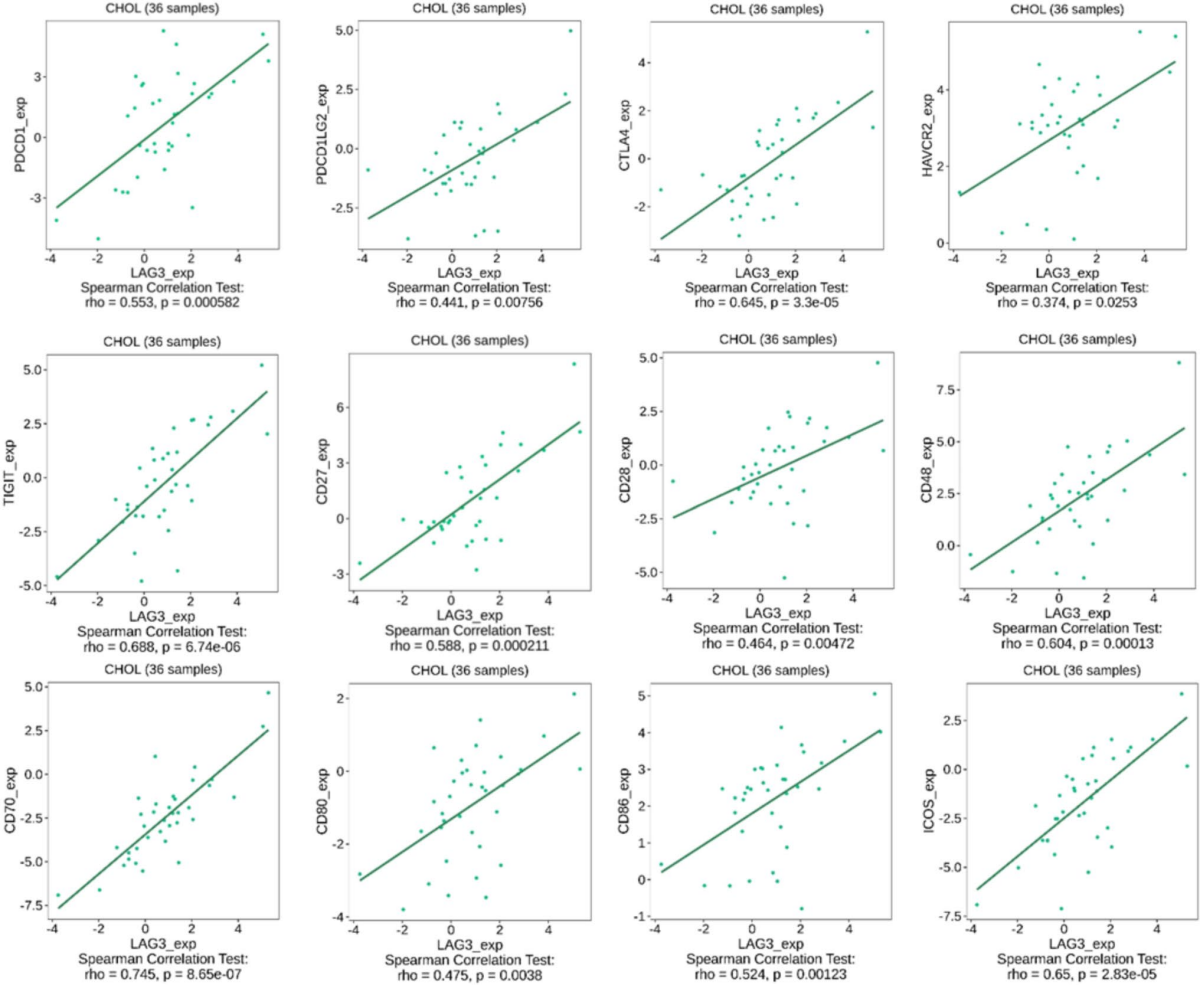
Correlation analyses between LAG-3 and other immune checkpoints expression in TCGA-CHOL datasets. Note. Correlation analyses of the RNA sequencing data extracted from the TCGA-CHOL dataset were conducted using the TISIDB web portal

**Fig. 7:**
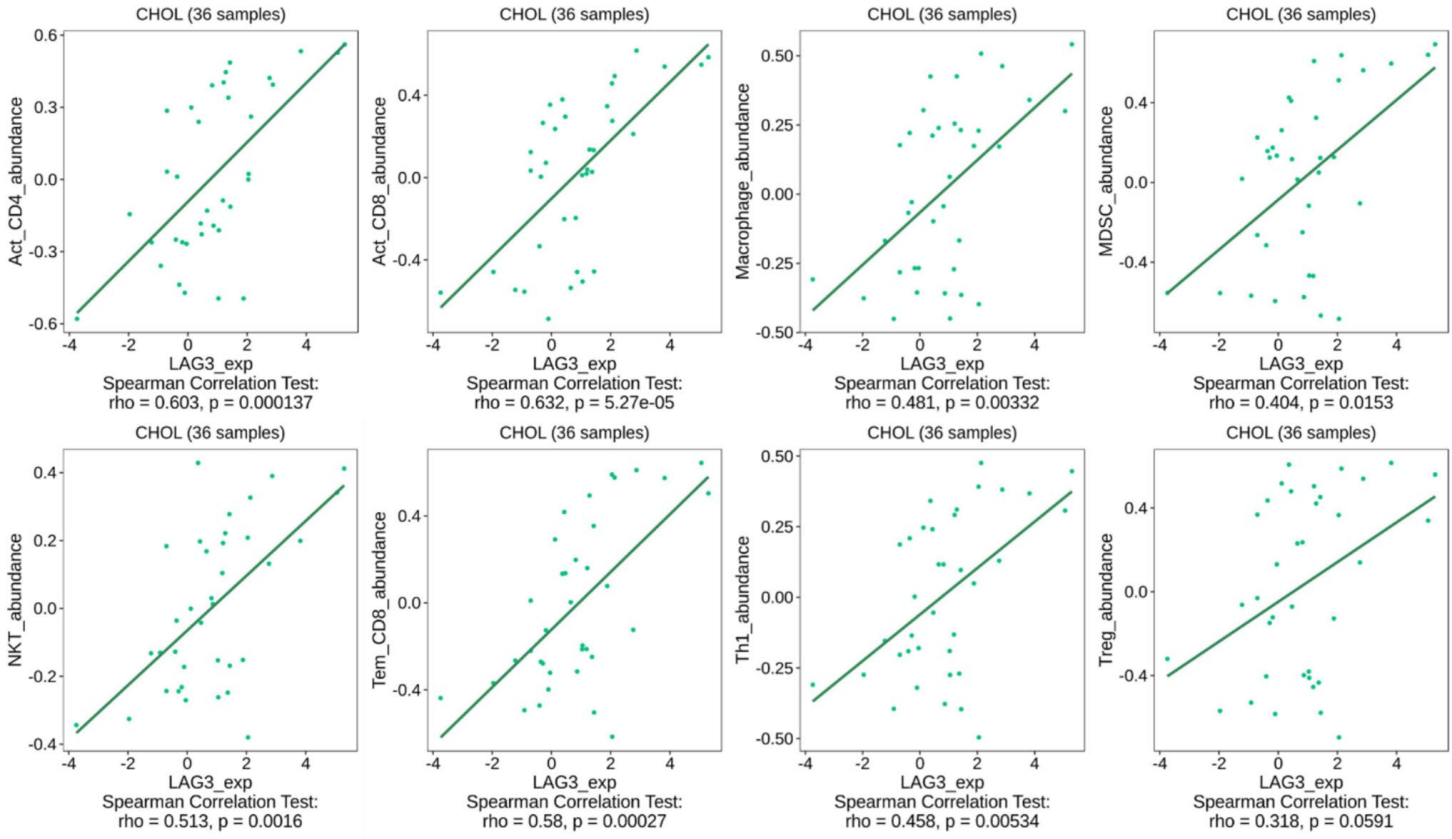
Correlation analyses between LAG-3 and tumor-infiltrated immune cells in TCGA-CHOL datasets by TISIDB web-portal. Note. The relative abundances of 28 tumor-infiltrating immune cell types, including CD4, CD8, helper T cells, regulatory T cells, B cells, myeloid-derived suppressor cells, natural killer T cells, dendritic cells, neutrophils, eosinophils, monocytes, and macrophages, were inferred using gene set variation analysis based on gene expression profiles. Abbreviations: Act_CD4, activated CD4^+^ T cells; Act_CD8, activated CD8^+^ T cells; MDSC, myeloid-derived suppressor cells; NKT, natural killer T cells; Tem_CD8, effector memory CD8^+^ T cells; Th1, T helper 1 cells; Treg, regulatory T cells

Therefore, we explored the TCGA-CHOL cohort to validate the dependence of LAG-3 expression on various co-stimulatory and co-inhibitory molecules. For the analysis, we recruited patients with TIGIT, CTLA-4, HAVCR2, PDCD1, PDCD1LG2, CD27, CD28, CD48, CD70, CD80, CD86, and ICOS expression. All co-stimulatory and co-inhibitory molecules revealed a positive correlation with LAG-3 in the TCGA cohort (Fig. 6). Moreover, LAG-3 expression moderately correlated with the infiltration of various immune cells into the tumor microenvironment, such as activated CD8^+^ T cells, effector memory CD8^+^ T cells, activated CD4^+^ T cells, T helper 1 cells, myeloid-derived suppressor cells, natural killer T cells, and macrophages (Fig. 7). Based on the above evidence, we concluded that LAG-3 plays a pivotal role in BTC and reprograms the immune population.

## Discussion

In this study, we identified LAG-3 expression as a valuable biomarker for predicting the clinical benefits of chemoimmunotherapy in ABTC. Notably, LAG-3 positivity correlated with a higher ORR and more profound tumor shrinkage. Furthermore, there was a positive correlation between the proportion of LAG-3-stained immune cells and both ORR and depth of tumor response. Patients in the LAG-3-positive subgroup exhibited longer PFS and OS. Although PD-L1 was substantially co-expressed with LAG-3, it did not serve as a predictive biomarker for ORR, PFS, or OS. LAG-3 expression enables the stratification of patients with ABTC into chemoimmunotherapy-sensitive and chemoimmunotherapy-insensitive subgroups, thereby aiding in the optimization of chemoimmunotherapy trial designs and refining treatment strategies.

Although PD-L1 expression is widely studied and considered a potentially useful biomarker in immuno-oncology, its effectiveness remains inconclusive in large phase III clinical trials for ABTC [24, 25]. Emerging evidence points to the potential of liquid biopsy-based biomarkers, gut microbiota, and genomic alterations as complementary predictors of immunotherapy response in BTC [26, 27]. LAG-3, which was initially identified in T and NK cells in 1990 [28], consists of three domains: immunoglobulin-like, transmembrane, and cytoplasmic. The Ig-like domain mediates interactions with MHC II, which is located on antigen-presenting cells, along with other LAG-3 ligands from tumor cells, hepatocytes, monocytes, and neurons. The transmembrane domain links the Ig-like and cytoplasmic domains and is cleaved by A Disintegrin and Metalloproteinases 10/17, releasing soluble LAG-3 with undefined functions. The cytoplasmic domain contains two motifs with known functions: the KIEELE motif, essential for IL-2 production; and the C-terminal EP motif, which disrupts the CD3-CD4/CD8 complex interaction with lymphocyte-specific protein tyrosine kinase, limiting phosphorylation of CD3ζ and Zeta-chain-associated protein kinase 70, thereby attenuating TCR signaling [29, 30]. The antitumor activity of cytotoxic LAG-3^+^ CD8^+^ T cells is generally reduced, and the expression of LAG-3 in regulatory T cells enhances their immunosuppressive functions and promotes tumor growth. Consequently, LAG-3 is considered as a marker of exhausted CD4^+^ and CD8^+^ T cells.

LAG-3 expression is correlated with poor prognosis in renal clear cell carcinoma (RCC), low-grade glioma, uveal melanoma, hepatocellular carcinoma (HCC), head and neck squamous cell carcinoma, and non-small cell lung cancer [31–33]. Conversely, LAG-3 expression is associated with a favorable prognosis in esophageal squamous cell carcinoma, resectable gastric cancer, and early breast cancer [34–36]. Heterogeneity in intratumoral LAG-3 expression may explain the complex relationship between LAG-3 expression and cancer prognosis. Carapeto et al. analyzed 100 tumor samples and observed a 40% LAG-3 positivity rate in BTC samples, which is similar to the 38% positivity rate observed in our study. They noted worse survival in patients with high PD-L1 and LAG-3 expression along with low CD3, CD4, and ICOS expression, especially in the tumor center [37]. Although previous studies have identified LAG-3 as a negative prognostic indicator, it is important to recognize that most study participants did not receive ICI treatment, leaving the prognostic value of LAG-3 for predicting ICI outcomes largely unexplored.

LAG-3 is co-expressed and engages in crosstalk with other immune checkpoints, including PD-1 and CTLA-4, in various cancer types [38]. This correlation is similar to that observed in our BTC study. Although LAG-3 expression did not correlate with PD-L1 expression in the TCGA-CHOL cohort, this discrepancy could be due to PD-L1's nonspecific expression on both tumor and immune cells, in contrast to LAG-3's specific expression on immune cells. Furthermore, we observed an enrichment of CD8^+^ T cells in LAG-3-positive BTC, which is consistent with previous findings in melanoma, where LAG-3-positive melanoma exhibited higher levels of CD8^+^ T cells and responded more effectively to treatments targeting either PD-1 alone or in combination with LAG-3 [39, 40]. Additionally, a biomarker study using CheckMate 040 identified a 4-gene inflammatory signature consisting of PD-L1, CD8A, LAG-3, and signal transducer and activator of transcription 1 associated with higher ORR and improved OS in ICI-treated HCC, suggesting that the presence of an inflammatory signature may indicate a tumor microenvironment with an abundance of exhausted T cells that could be more amenable to ICI therapy [41]. Job et al. identified four immune subtypes of IHCC, with 11% categorized as the immune-inflamed subtype, marked by enriched T cell infiltration and activated immune checkpoint pathways [7].

The interaction between PD-1/PD-L1 and LAG-3 forms the basis for the combination of anti-LAG-3 and anti-PD-1/PD-L1 therapies to achieve synergistic effects. In RCC, LAG-3 expression is significantly upregulated following PD-1 inhibition. Furthermore, a notable increase in interferon-gamma-positive CD8^+^ T cells was observed after concurrent blocking of LAG-3 and PD-1, compared to the inhibition of PD-1 alone [42]. Serum levels of LAG-3 in patients with melanoma increase after anti-PD-1 monotherapy and subsequently decrease following dual blockade of LAG-3 and PD-1 [40]. This indicates that anti-LAG-3 therapy could mitigate the compensatory upregulation of LAG-3 triggered by PD-1 inhibition. Consequently, dual inhibition of LAG-3 and PD-1 has demonstrated significant improvements in the clinical outcomes of melanoma [43]. Interestingly, the effectiveness of anti-LAG-3 therapy appeared to be independent of LAG-3 expression levels. Tumors that exhibit an increase in post-treatment effector CD8^+^ T cells reveal a better therapeutic response [17, 44]. Given the effectiveness of anti-LAG-3 in melanoma, there is a growing need for additional clinical trials to explore the combination of anti-PD-1/PD-L1 and anti-LAG-3 in different cancer types. Our findings highlight potential synergies between anti-PD-1 and anti-LAG-3 inhibitors in ABTC. Therefore, the combination of chemotherapy with Opdualag, a premixed combination of anti-LAG-3 and anti-PD-1 monoclonal antibody, could be explored as a first-line treatment for advanced BTC in future clinical trials.

Chromatin remodeling genes, including those encoding switch/sucrose non-fermentable (SWI-SNF) complexes, have been shown to regulate genomic architecture and influence responses to ICIs in prior research [45]. In our previous study, loss-of-function (LoF) mutations in chromatin remodeling genes were identified as significant predictors of prolonged PFS and OS in ABTC patients undergoing chemoimmunotherapy [19]. In the present study, we analyzed the correlation between oncogenic chromatin remodeling gene mutations and LAG-3 expression. A trend was noted toward higher LAG-3 positivity in tumors with LoF mutations (43% vs. 19%) compared to wild-type, though this difference did not achieve statistical significance (*P* = 0.15; data not shown). Currently, the correlation between LAG-3 expression and LoF mutations in chromatin remodeling genes in BTC has not been reported. Therefore, further investigation into the interactions and causal relationships between LoF mutations in chromatin remodeling genes and immune inhibitory signaling is warranted.

Over the next 5 years, biomarker research in ABTC immunotherapy is anticipated to evolve from single-molecule to multi-omics approaches, incorporating genomic, transcriptomic, epigenomic, proteomic, metabolomic, and microbiome data. The integration of machine learning tools is expected to enhance the precision of identifying patients likely to benefit from immunotherapy. Furthermore, these multi-omics strategies may provide deeper insights into resistance mechanisms, facilitating the development of novel therapeutic approaches to address these challenges and improve patient outcomes.

This study has several limitations. First, the majority of tumor samples were obtained via biopsy rather than surgical resection, which restricts our ability to fully evaluate intratumoral heterogeneity in LAG-3 expression and its impact on prognosis and treatment outcomes. Additionally, the relatively small sample size of 44 patients and the uniform treatment regimen of nivolumab combined with modified GS limit the generalizability of our findings to the broader BTC patient population, who may receive different treatments, such as anti-PD-L1 therapies or GC. As a post-hoc biomarker analysis, our study may be subject to biases inherent in retrospective analyses. Although a correlation between LAG-3 expression and treatment response was observed, the underlying mechanisms are not yet clear. Further research is needed to elucidate the biological role of LAG-3 in BTC.

In conclusion, our findings suggest that LAG-3 expression may serve as a valuable biomarker for identifying immune-inflamed BTC subtypes and predicting the response to chemoimmunotherapy in ABTC. Dual inhibition of PD-1/PD-L1 and LAG-3 may be a synergistic approach for the treatment of ABTC.

## Data Availability

The data that support the findings of this study are available on request from the corresponding author. The data are not publicly available due to privacy or ethical restrictions.

## Abbreviations

AEs: Adverse events
AVC: Ampulla of vater cancer
B7-H3: B7 homolog 3 protein
BTC: Biliary tract cancer
BTLA: B- and T-lymphocyte attenuator
BRCA: Breast cancer gene
CPS: Combined positive score
CTCAE: Common terminology criteria for AEs
CTLA-4: Cytotoxic T lymphocyte associated protein 4
DCR: Disease control rate
DSP: Digital spatial profiler
EHCC: Extrahepatic cholangiocarcinoma
FGFR: Fibroblast growth factor receptor
GBC: Gallbladder cancer
GC: Gemcitabine plus cisplatin
GITR: Glucocorticoid-induced TNFR-related protein
GS: Gemcitabine plus S-1
HAVCR2: Hepatitis A virus cellular receptor 2
HCC: Hepatocellular carcinoma
IHCC: Intrahepatic cholangiocarcinoma
ICIs: Immune checkpoint inhibitors
ICOS: Inducible T cell co-stimulator
IDH1: Isocitrate dehydrogenase 1
IHC: Immunohistochemistry
LAG-3: Lymphocyte activation gene 3
LoF: Loss-of-function
MHC: Major histocompatibility complex
NK: Natural killer
ORR: Objective response rate
OS: Overall survival
PD-1: Programmed death 1
PDCD1: Programmed cell death 1
PDCD1LG2: Programmed cell death 1 ligand 2
PD-L1: Programmed death ligand 1
PFS: Progression-free survival
RCC: Renal clear cell carcinoma
RECIST: Response evaluation criteria in solid tumors
ROIs: Regions of interest
SWI-SNF: Switch/sucrose non-fermentable
TIGIT: T cell immunoreceptor with immunoglobulin and immunoreceptor tyrosine-based inhibitory motif domains
TIM-3: T cell immunoglobulin and mucin domain-containing protein 3
U.S. FDA: U.S. food and drug administration
